# Metabolic scope, performance and tolerance of juvenile European sea bass *Dicentrarchus labrax* upon acclimation to high temperatures

**DOI:** 10.1371/journal.pone.0272510

**Published:** 2022-08-12

**Authors:** Orestis Stavrakidis-Zachou, Konstadia Lika, Michail Pavlidis, Mohamed H. Asaad, Nikos Papandroulakis

**Affiliations:** 1 Department of Biology, University of Crete, Heraklion, Crete, Greece; 2 Institute of Marine Biology, Biotechnology and Aquaculture, Hellenic Centre for Marine Research, Heraklion, Crete, Greece; 3 Beacon Development, King Abdullah University of Science and Technology, Thuwal, Saudi Arabia; COISPA Tecnologia & Ricerca - Stazione Sperimentale per lo Studio delle Risorse del Mare, ITALY

## Abstract

European sea bass is a species of great commercial value for fisheries and aquaculture. Rising temperatures may jeopardize the performance and survival of the species across its distribution and farming range, making the investigation of its thermal responses highly relevant. In this article, the metabolic scope, performance, and tolerance of juvenile E. sea bass reared under three high water temperatures (24, 28, 33°C), for a period of three months was evaluated *via* analysis of selected growth performance and physiological indicators. Effects on molecular, hormonal, and biochemical variables were analyzed along with effects of acclimation temperature on the metabolic rate and Critical Thermal maximum (CT_max_). Despite signs of thermal stress at 28°C indicated by high plasma cortisol and lactate levels as well as the upregulation of genes coding for Heat Shock Proteins (HSP), E. sea bass can maintain high performance at that temperature which is encouraging for the species culture in the context of a warming ocean. Critical survivability thresholds appear sharply close to 33°C, where the aerobic capacity declines and the overall performance diminishes. European sea bass demonstrates appreciable capacity to cope with acute thermal stress exhibiting CT_max_ as high as 40°C for fish acclimated at high temperatures, which may indicate resilience to future heatwaves events.

## Introduction

European sea bass (*Dicentrarchus labrax*, Linnaeus, 11758) is a species of great economic importance and constitutes, along with gilthead seabream (*Sparus aurata*, Linnaeus, 1758), the main farmed fish in the Mediterranean with an annual production exceeding 200,000 tonnes [1]. The species is eurythermal and known to have great adaptation capacity to rapidly fluctuating temperatures [2]; traits which have contributed to its widespread distribution and commercial exploitation. However, it is uncertain whether the production of such a key species will remain secure in the future in the context of global warming, with studies postulating on the potential effects of climate change on the species and the overall aquaculture production across its distribution range [3–5]. Depending on the region and choice of climate scenario, such effects may include, among others, positive or negative changes in growth performance, seasonal shifts in growth patterns, adverse impacts of extreme events on survival and growth, changes in the physiology and health status of the fish, as well as changes in production volumes and the profitability of the farms [3–8]. In that regard, assessing the vulnerability of E. sea bass to climate change necessitates consideration of both chronic and acute effects of elevated temperatures on its physiology. The former reflect gradual shifts to higher temperature regimes due to the overall rising of ocean temperatures while the latter represent acute warming events such as heatwaves whose duration lasts from hours to days [9].

For E. sea bass, the need to address existing knowledge gaps regarding its response to higher temperature regimes as well its tolerance during acute thermal stress is highlighted by reports of seawater temperature already exceeding 30°C during heatwaves in some coastal areas where the species is farmed [10], which are thought to be close to the species thermal limits [11]. Moreover, with typical winter and summer Sea Surface Temperatures (SST) ranging between 13–17°C [12] and 24–28°C [13] respectively, the Mediterranean Sea has already been recognized as a particularly vulnerable area to climate change and climatic projections predict an alarming temperature increase in the coming decades [14–16]. Specifically, analyses on an ensemble of downscaled climatic model outputs for the region suggest potential increase of 1.9–3.8°C (depending on the choice of climate scenario) by the end of the century compared to the reference period of 1981–2010 [17]. Such an increase may translate to SST of 29–31°C for the western part of the Mediterranean basin while maximum temperatures as high as 33°C may be exhibited in the eastern parts during heatwaves [18]. In fact, areas such as the northwest Ionian, the Balearic islands, and the Aegean and Levantine Seas have been identified as particularly sensitive [19] with predictions for the increase of summer SST being up to 5°C compared to today’s values [18] under the most pessimistic, but highly likely [20], climate scenarios.

With respect to the chronic exposure to a stressor such as high temperature, fish typically undergo an acclimation process during which, substantial molecular, biochemical, and morphological changes may occur [21,22]. Depending on the species acclimation capacity this process may require several weeks or months and thus it is vital to study the timescales at which acclimation occurs [23]. Evaluation of these changes involves not only the quantification of performance indicators typically used in fish research such as the growth rate and the Feed Conversion Ratio (FCR) but also the determination of various physiological indicators related to thermal stress. For instance, plasma concentrations of hormonal such as cortisol and biochemical parameters such as glucose, lactate, cholesterol, and triglycerides exhibit shifts as a response to temperature changes and are often used to assess the health status of the fish at different temperatures [24–29]. Moreover, changes in the metabolic rate via measurement of the respiration rate and determination of the Standard Metabolic Rate (SMR) and the aerobic scope may be used to interpret temperature effects on the energetic demands [30–32] while shifts in the relative expression of specific genes like those encoding stress-related proteins such as the Heat Shock Proteins (HSP) may be useful in assessing the molecular response to stress of the fish [33,34]. Furthermore, large scale organismal modifications may even be reflected by changes in the relative organ size such as for instance the enlargement of the heart (heart hypertrophy) which has often been associated with hypoxia or high temperatures [35].

On the other hand, the capacity of fish to cope with acute thermal challenges has been typically evaluated by determination of critical temperatures [36–39]. In this context, the Critical Thermal maximum (CT_max_), which represents the temperature at which physiological failure occurs under an acute thermal challenge, is often determined to quantify the upper tolerance limits. While CT_max_ tends to increase with acclimation temperature, the magnitude of this trend varies considerably among species and for that reason, the Acclimation Response Ratio (ARR) may also be reported. The AAR is a convenient index that describes the magnitude of thermal acclimation and allows for inter-species comparisons, with stenothermal species living in either cold or warm environments typically exhibiting lower values than those subjected to regular temperature fluctuations [40,41].

For E. sea bass, there is a large body of research regarding its thermal responses, both chronic and acute, to a wide range of temperatures; yet, critical information towards the upper edge of the species tolerance range is missing. In its natural habitat E. sea bass is exposed to temperatures typically ranging from 6 to 28°C [40] where the positive correlation of temperature with growth, feed intake, digestion speed, and feed conversion efficiency has been well documented [42–44]. Further experimentation at temperatures between 6–30°C, has also provided insights regarding the width of the thermal tolerance of the species [44–47]. Specifically, Person-Le Ruyet *et al*. [44], report that the growth, nitrogen excretion and oxygen consumption rates reach maximum values at 26–27°C before deteriorating, thus indicating the thermal optimum for the species. Finally, recent efforts such as those of Islam *et al*. [26,27,46] are exploring effects of acclimation temperatures as high as 32°C on E. sea bass fingerlings (5 g). Yet, the existing studies at the upper end of the tolerance range are restricted to small fish and short time-scales ranging from a few days to up to a month, with limited information on the performance, health, physiological status and tolerance of E. sea bass. Therefore, in the prism of global warming it is evident that further experimentation is needed to elucidate the responses of E. sea bass on high temperatures which will not only corroborate previous findings but also address existing knowledge gaps.

In this article, we present the results from a thermal trial conducted at the Hellenic Center for Marine Research using juvenile E. sea bass. The objective was to study the responses of the species under high acclimation temperatures and determine its tolerance under acute thermal stress since both chronic and acute time-scales are relevant for aquaculture in the context of climate change. Specifically, we tested three acclimation temperatures, namely 24, 28 and 33°C, and determined shifts in a range of indicators aiming to link to the whole-animal performance. The selection of experimental temperatures was done in order to explore thermal effects at relevant ecological and climatic scales. The lowest temperature (24°C) represents a typical summer/autumn temperature [18] exhibited throughout the farming distribution of the species and can therefore act as a reference point for further comparisons. The intermediate temperature (28°C) represents the highest temperatures currently recorded at certain parts of the Mediterranean [18,48] and, thus, may provide insights into potential thermal effects anticipated within the next few years. Finally, the highest temperature (33°C) was used to postulate on thermal effects at hypothetical, yet ecologically relevant, scales projected by climate change within the next decades [17–19].

Unlike most existing studies for the species which focus on short-term effects at the edges of the tolerance range, the duration of the trial was set at three months, allowing sufficient time for assessing the prolonged effects of temperature on the selected indicators and the fish performance [29,49,50]. In addition, taking into account that most published work on the species at high temperatures is limited on fingerlings, we here used bigger juveniles of intermediate size (150g). This size coincides with the grow-out stage in commercial aquaculture, which predominantly takes place in marine cages, and is therefore relevant in the context of climate change since the fish are exposed to coastal environmental conditions. A final objective of our study was to investigate the capacity of the species to cope with acute thermal stress, which was done via the determination of the CT_max_. In that regard, the hypothesis that this capacity correlates positively with the thermal history (acclimation temperature) of the fish was also tested.

## Materials and methods

### Experimental design

This study was performed at the land-based facilities of the Institute of Marine Biology, Biotechnology, and Aquaculture (IMBBC) in Crete, in certified laboratories (EL91‐BIOexp‐04) in accordance with legal regulations (EU Directive 2010/63) and after its approval by the Ethics Committee of the IMBBC and the regional veterinary authorities (Department of Veterinary Services, Ref Number 255, 344).

The system used in the trial (performed between Apr and Jul 2019) included three separate thermoregulated marine RAS (Recirculating Aquaculture System) which were used for the different temperature treatments. Each RAS comprised of three cylindroconical tanks (2 m^3^), that acted as replicates (three replicate tanks per treatment, nine in total), connected to a biological and a mechanical filter. In order to select appropriate sample size for the trial, an initial power analysis was conducted using the G*Power software (version 3.1.9.7) which indicated a number of 30 fish per tank as sufficient for detecting effects of medium magnitude (effect size, f = 0.25) at a power of 0.8. However, in anticipation of high temperature-related mortalities as found in previous work [51] as well as considering fish removals due to sampling the actual number of fish used in this study was doubled.

Fish stock for the trial was obtained from the institute’s pilot scale cage farm (Souda Bay, Crete) and is of Eastern Mediterranean broodstock origin. Upon arrival to the laboratory, the fish were maintained in a holding tank for a quarantine and acclimation period of three weeks during which they were subjected to an antiparasitic treatment (150 mg L^-1^ formalin bath for 45 minutes). Regarding the subsequent distribution of the fish to the experimental tanks, a block randomization procedure was applied. Specifically, lists containing numbers one through nine were randomized in excel. Fish were then caught with a net from the holding tank, anesthetized (2*-phenoxyethanol bath*, *0*.*2 mg L*^-1^) in groups of ten, measured individually for length and weight and then transferred to the corresponding tank of the list until all tanks received the first batch. This process was repeated for a total of seven times (for the last one only two fish were distributed in each tank), ensuring that all ranks received the same number of fish (62). The initial weight and total length of the fish was 135.3 ± 1.9 g and 23.3 ± 0.8 cm respectively while individuals that exhibited skeletal abnormalities and other deformities such as missing or damaged fins and eyes were excluded from the trial. Finally, it’s worth noting that while the allocation of tanks to treatments was predetermined based the facilities design, this information was not revealed to the personnel handling the fish during the distribution to avoid potential bias. Due to limited human resources no further blinding procedure was applied at later stages of the trial.

Upon the start of the trial, temperature in each treatment was raised at a rate of 1°C per day until the experimental temperatures of 24, 28 and 33°C (forming the T-24, T-28, and T-33 treatments) were reached. The variability of temperature was around 1°C at each treatment throughout the trial, which lasted three months. During that time, sampling for growth performance as well as biochemical indicators was performed on a monthly basis, as detailed in the next paragraph, leading to a total of three samplings for each treatment (at 30, 60 and 90 days). The determination of CT_max_ was performed immediately after the 90 day mark, and fish completing the acute thermal challenge were transferred to a holding tank for recovery. This was followed by the measurement of the metabolic rate which ensured that the fish used for respirometry were different from those used for CT_max_ determination as well as those used for the biochemical and genetic analyses.

Throughout the trial, the tanks were inspected visually for mortalities several times per day and dead fish were removed immediately and recorded. Moreover, in order to minimize suffering, any fish found in moribund state or displayed erratic swimming behavior coupled with partial or complete loss of equilibrium were also removed and euthanized according to veterinary guidelines [52] by immersion to a euthanasia bath (2*-*phenoxyethanol 0.6 mg L^-1^) for at least ten minutes following cessation of opercular movement.

### Fish rearing and sampling

Feeding was performed to satiation by hand twice per day using a commercial feed for E. sea bass (45% crude protein, 16% crude fat; provider: IRIDA S.A., Greece). Any uneaten feed was collected from the bottom of the tanks and weighed at the end of the day which allowed for the calculation of the daily feed intake (DFI) expressed as percentage of fish body weight (% BW d^-1^). Tanks were equipped with automatic oxygen sensors for continuous monitoring of dissolved oxygen and temperature, and oxygen was supplied directly to the tanks when needed in order to maintain oxygen saturation above 80%. In addition, water quality parameters such as nitrogenous compounds (nitrate, nitrite, and total ammonia nitrogen), salinity and pH were monitored on a weekly basis via manual measurements and kept within the standard safe limits.

Regarding sampling, in each month all fish were anesthetized and their weight and total length was individually measured. The individual measurements from all fish in each tank were used for subsequent calculations of mean weight and growth performance indices. Moreover, five fish per replicate (N = 15, per treatment) in each sampling were used for the determination of biochemical parameters in the plasma. Specifically, upon collection of blood, those fish were also sacrificed for tissue collection by emersion to a euthanasia bath as described previously. Blood was collected from the caudal vein using heparinised syringes within 10 min from fish being anesthetized, it was centrifuged (10 minutes, 2000g), and the plasma was stored at -20°C until further analysis. With respect to tissues, liver and heart were weighed (at 0.01g precision) and samples from liver and spleen were stored at -80°C after freezing in liquid nitrogen.

### Growth performance and somatic indices

The growth performance as well as changes in relative organ size of E. sea bass were assessed via calculation of the corresponding parameters below. Specifically, for each of the three sampling months, the Absolute Growth Rate (AGR) (g d^-1^) was calculated as:

$$AGR=\frac{M{BW}_{2}-{MBW}_{1}}{t_{2}-t_{1}}$$

where *t*_1_ and *t*_2_ denote the time (days) at the beginning and end of the specific month since the start of the trial, and *MBW*_1_ and *MBW*_2_ are the respective mean (at tank level) whole body weights of the fish at those times. For the calculation of the mean weights, the individual measurements of all fish in each tank were taken into account.

With respect to feeding, the feed eaten (g) at the tank level over each sampling period was recorded (tank feed intake, *TFI*). Therefore, the feed intake (*FI*) per individual was calculated as:

$$FI=\frac{TFI}{\frac{N_{1}+N_{2}}{2}}$$

with *N*_1_ and *N*_2_ being the number of fish in the tank at times *t*_1_ and *t*_2_, while the daily feed intake (*DFI*) expressed in percentage of weight (% BW d^-1^) as:

$$DFI=100\frac{FI}{\left(\frac{{MBW}_{1}+M{BW}_{2}}{2})(t_{2}-t_{1}\right)}$$

Accordingly, the Feed Conversion Ratio (FCR) was calculated as:

$$FCR=\frac{FI}{{MBW}_{2}-M{BW}_{1}}$$

while for *N*_*d*_ being the number of fish the died within each period and *N*_0_ the initial population, the survival (S) at the tank level was calculated as:

$$S=100(1-\frac{N_{d}}{N_{0}})$$

Regarding the somatic indices, at the three sampling points the Hepatosomatic (HSI) and Cardiosomatic (CSI) indices were calculated for each fish as:

$$HSI=100\;\frac{LW}{BW}$$

$$CSI=100\;\frac{HW}{BW}$$

where *BW*, *LW*, *HW* are the whole body, liver, and heart weight (g) of the fish.

### Metabolic rate determination

The metabolic rate of E. sea bass was measured *via* respirometry. Specifically, the Standard (SMR) and the Maximum (MMR) Metabolic Rates were determined for the three treatments and the Absolute Aerobic Scope (the difference between SMR and MMR) and the Factorial Aerobic Scope (MMR divided by SMR) were calculated. An intermittent flow respirometer was used (Loligo Systems) which comprised of four individual 2 L metabolic chambers. Each chamber was connected to a dipping probe mini oxygen sensor which was calibrated (two point calibration using Na_2_SO_3_ solution and air-equilibrated water sample for 0 and 100% respectively) prior to the measurements. For each temperature treatment, the system was connected to the respective RAS which ensured that constant temperatures were maintained during the measurements.

The two metabolic rates were determined according to established protocols [30,53,54]. Briefly, the fish were first placed in a separate circular tank (80 L) where they could swim unimpeded and an exhaustive protocol was applied. The protocol involved chasing the fish with a net until they lost capacity for burst swimming, which typically lasted between 2–5 minutes. They were then taken out of the tank, which was positioned next to the respirometer, and immediately placed in the metabolic chambers. The duration of this procedure was less than 20 seconds, thus minimizing air-exposure of the fish. A recording of oxygen consumption rate was taken over the first three minutes as a proxy for representing the MMR. Subsequently, the fish were left in the respirometer for a period of 24 hours and recordings where taken at 10 minute intervals. The duration of flush period (at 5 L m^-1^) was set at six minutes followed by one minute of waiting between recordings which ensured complete renewal of the water within the chambers and stabilization of the oxygen levels prior to the measurement. Measurements corresponding to slopes with a regression coefficient (r^2^) lower than 0.95 were discarded from the analysis and the remaining were used to calculate the SMR using the q0.2 quantile method as described in Chabot *et al*. [30]. The measurements were also adjusted to a body mass of 200 g using a mass exponent of 0.77 [45]. The oxygen saturation within the chambers was not allowed to fall below 80% while the exhaustive protocol and the duration of the flush cycle described above were calibrated from preliminary trials in our lab.

To account for background respiration, a single recording of 20m duration was taken on each chamber before and after the measurements. Because the before and after values did not differ substantially, a time-dependent background respiration correction was not applied but instead the average between the before and after measurements was subtracted from the rest of the recordings. In the study of SMR, it is recommended that experimenters try to minimize the effect of post-prandial metabolism. This in turns requires knowledge of the species-specific gut evacuation time which depending on the species, temperature, and feed composition may last several days [30]. Considering that for E. sea bass complete gut evacuation may last as long as two days [55] the fish in this study were fasted for 48 h before the measurement. Furthermore, external stimuli were minimized by visually isolating the fish from their surrounding with the help of a black curtain. The measurements were performed at the end of the trial on 15 fish per treatment while the chambers and the related tubing was cleaned with a mild bleach solution between treatments.

### Acute thermal challenge

The tolerance of E. sea bass to temperature extremes was evaluated by performing an acute thermal challenge upon completion of the three month trial. In this challenge, the Critical Thermal Maximum (CT_max_) and the resistance time were determined for the three acclimation temperatures while the Acclimation Response Ratio was calculated to assess the acclimation capacity of E. sea bass among treatments. For the determination of CT_max_, we applied a similar protocol described in Dülger *et al*. [40] and Kir *et al*. [39]. Briefly, fish were placed individually in an insulated 50 L container filled with water of their respective temperature treatment. Subsequently, the temperature was increased at a rate of 0.5°C min^-1^ by means of slow flow of hot water from a reservoir placed above the container according to Chrétien and Chapman [56]. An overflow ensured that the water level was maintained constant throughout the challenge while temperature and oxygen monitoring was done with an LDO sensor (Hach Lange GmbH, Germany) and the temperature at which the endpoint was reached was recorded. The endpoint was set as the temperature at which fish lost their dorso-ventral orientation, also known as the point of Loss of Equilibrium (LoE) [38]. Upon reaching the LoE, each fish was transferred back to a holding tank for recovery. Apart from two fish from the highest temperature treatment no other mortalities were recorded following this challenge. For each treatment 15 fish (five per replicate) were used in total and the CT_max_ was defined as the mean temperature at which the endpoint was reached. Similarly, the mean time required to reach the endpoint was recorded as the resistance time. Finally, the ARR was calculated by dividing the change in tolerance (CT_max_) between acclimation temperatures by the change in acclimation temperature according to Claussen [57].

### Analytical procedures

Commercial kits (BIOSIS Ltd., Greece; Sigma-Aldrich, Germany) were used to measure via spectrophotometric methods the biochemical parameters in plasma, namely the concentrations of triglycerides (mmol l^-1^) and lactate (mmol l^-1^). Moreover, the plasma cortisol concentrations (ng ml^-1^) were quantified using a previously validated [58] enzyme immunoassay kit (DRG International, Germany).

### Gene expression

A molecular analysis was performed to study the expression of three target genes, namely those coding for the Heat Shock Proteins HSP70 and HSP90, and the Glucocorticoid Receptor (GR). For each treatment, the analysis was performed in two tissues (spleen and liver) from samples taken from five individuals (five per treatment). The focus of this analysis was to assess long-term thermal effects and hence, it was performed at the end of the trial. The primers were described by our group (Dr A. Tsalafouta, Laboratory of Fish Physiology) for the target genes GR (FWD: 5’-GAGATTTGGCAAGACCTTGACC-3’; REV: 5’- ACCACACCAGGCGTACTGA-3’), HSP70 (FWD: 5’- GATGAAGGAGATCGCCGAAGCC-3’; REV: 5’-GGCCTGTCGCTGGGAGTC-3’), and HSP90 (FWD: 5’- GCCTCTGATGCTTTGGAC-3’; REV: 5’- GCTTTGTTGGGGATGATGT-3’). The expression of the target genes was calculated as relative to that of a reference gene via quantitative real-time Polymerase Chain Reaction (qPCR). The reference gene was β-actin, which was selected according to Vandesompele *et al*. [59] among three candidate genes (β-actin, ribosomal RNA S18, and eEEF1-α) as it exhibited the most stable expression among samples. A qPCR thermocycler (CFX connect real-time, Bio-rad) was used for the analysis which was performed according to the manufacturer’s instructions using KAPA SYBR FAST Universal (KAPA Biosystems) kits. Specifically, the steps for the qPCR involved an initial denaturation for three minutes (95°C), followed by 35 cycles (15 sec at 95°C, 30 sec at 60°C, 2 sec at 72°C), and finally a melt-curve performed with a half degree increment from 65°C up to 90°C. Serial dilutions of pooled cDNA samples (1:5, 1:25, 1:125, 1:625) were used to construct a standard curve for each gene.

Claussen [57].

### Statistical analysis

The effect of temperature over time on the growth parameters, namely the AGR, FI and FCR, was analysed by constructing Linear Mixed Models (LMMs) which were fitted through restricted maximum likelihood. These models constitute appropriate tools for dealing with the non-independency of data such as, for instance, repeated measures on the same experimental unit [60]. Specifically for our trial, in order to account for the repeated measures on each tank we used the tank ID as a potential random effect. The temperature, time and their interaction were treated as fixed effects. In addition, the marginal and conditional ***R***^2^ ($R_{m}^{2}$ and $R_{c}^{2}$ respectively) were computed for each model according to Nakagawa and Schielzeth [61]. The $R_{m}^{2}$ refers to the variance explained by the fixed effects while the $R_{c}^{2}$ to the variance explained by the fixed and the random effects, and therefore their difference represents the variability among experimental units. Regarding the biochemical variables and the somatic indices we used analysis of variance (two-way nested ANOVA) to evaluate the effects of temperature and experimental duration on the physiological indicators. For the metabolic rate determination, the relative gene expression, the acute thermal challenge, as well as the survival at the end of trial, a one-way ANOVA was performed. The level of significance for all analyses was set at a *P* < 0.05 and the assumption criteria were checked using the Levene’s test for homogeneity of variance and the Kolmogorov-Smirnov for normality. For determining differences between groups the Tukey’s multiple comparisons test was performed. The statistical software SPSS (version 22) was used.

## Results

### Growth performance and somatic indices

**Fig 1:**
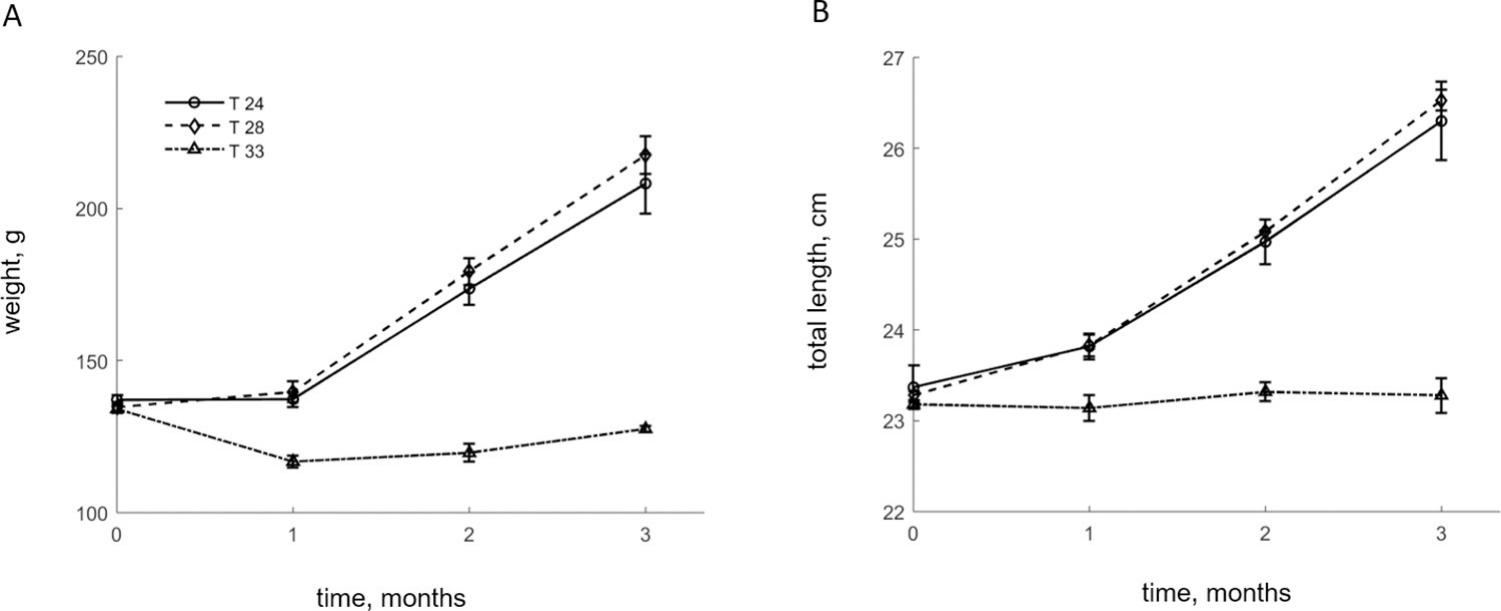
Weight (A) and total length (B) for E. sea bass reared under three temperatures (T-24, T-28, T-33). Points denote mean values between replicates and bars the standard deviation.

Temperature and time had significant effects on most of the considered growth performance indicators while significant interactions were also found between temperature and time. Overall, growth performance was similar for treatments T-24 and T-28 while it was markedly lower for T-33 throughout the trial (Table 1). Moreover, E. sea bass showed signs of slow acclimation to the changing temperature regimes with the growth performance being poor for all treatments during the first month (Table 1, Fig 1).

**Table 1:** Growth performance indicators for E. sea bass reared under three different water temperatures (T-24, T-28, T-33). AGR: Absolute Growth Rate; DFI: Daily Feed Intake; FCR; Feed Conversion Ratio. Values represent the mean and the SD among replicates (N = 3). Statistically significant differences between treatments in each sampling (month) are indicated with different letters and between samplings for each treatment with different numbers.

	1^st^ month				2^nd^ month			3^rd^ month	
	T-24	T-28	T-33	T-24	T-28	T-33	T-24	T-28	T-33
AGR (g d^-1^)	0.0 ± 0.1 ^a,1^	0.1 ± 0.1^a,1^	-0.4 ± 0.1^b,1^	1.2 ± 0.2^a,2^	1.3 ± 0.1^a,2^	0.1 ± 0 ^b,2^	1.1 ± 0.1^a,2^	1.3 ± 0.1^a,2^	0.3 ± 0.1^b,2^
DFI (% BW d^-1^)	1.1 ± 0.1^a,1^	1.1 ± 0.1^a,1^	0.9 ± 0.1^a,1^	1.3 ± 0.1^a,2^	1.5 ± 0.1^b,2^	0.8 ± 0.0^c,2^	1.3 ± 0.1^a,2^	1.4 ± 0.1^a,2^	1.0 ± 1^b,1^
FCR	7.5 ± 1.2^a^	6.4 ±4 .2^a^	-2 ± 0.6^b^	1.7 ± 0.3^a^	1.9 ± 0.2^a^	13.7 ± 5.6 ^b^	2.0 ± 0^a^	1.9 ± 0.1^a^	7.1 ± 2.6^b^

The daily feed intake (DFI) was minimally affected by the random effect (tank) while the fixed effects accounted for most of the variance ($R_{m}^{2}$: 0.49, $R_{c}^{2}$: 0.53). Specifically, for the resulting LMM, the random effect was found insignificant (*wald **χ***^2^ = 0.59; *P* = 0.561) while that of the fixed effects were significant (temperature: *F_2,16_* = 83.1, *P <* 0.001; time: *F_2,16_* = 21.4; *P* < 0.001; interaction: *F_4,16_* = 13.8; *P* = 0.004). During the temperature increase stage, feed intake remained low at approximately 0.5% BW d^-1^ for all treatments. However once the experimental temperatures were reached (week one of experiment) it progressively increased, exceeding 1% BW d^-1^ by the time of the first monthly sampling (Table 1). While it increased further for T-24 and T-28 over the next months, it remained consistently lower for T-33. Growth, expressed via the AGR, was similarly affected. In particular, the random effect was not significant (*wald **χ***^**2**^ = 0.36; *P* = 0.713) and explained a small fraction of the variance compared to the fixed effects ($R_{m}^{2}$: 0.76, $R_{c}^{2}$: 0.82), which significantly affected the AGR (temperature: *F_2,16_* = 124.7, *P* < 0.001; time: *F_2,16_* = 158.1; *P* < 0.001; interaction: *F_4,16_* = 8.2; *P* = 0.001). In response to the low feed consumption, growth was negligible during the first month for T-24 and T-28 which reached average weights of 137.3 ± 2.5 and 139.7 ± 3.4 g respectively (Fig 1, Table 1). Conversely, substantial weight loss was observed in T-33 which exhibited an average value of 116.8 ± 1.9 g. However, during the second month fish grew appreciably at the T-24 and T-28 treatments reaching weights of 173.7 ± 5.4 and 179.7 ± 4.3 g respectively, although the differences between the groups were not significant as indicated by the Tukey’s multiple comparisons test. Fish in T-33 exhibited negligible growth at the second sampling. Weight increased further in the third month for T-24 and T-28 with no differences between the groups, reaching maximum weights of 208.3 ± 9.8 and 217.5 ± 6.3 g respectively. As for T-33, the growth rate remained unsubstantial, yet positive (AGR, 0.3±0.1 d^-1^) in the third month. The same trend was observed for length of all treatments throughout the trial (Fig 1). Regarding the FCR, the only significant effect detected by the LMM was that of the interaction between temperature and time (*F_4,16_* = 14.9; *P* = 0.001). In the first sampling, the low growth of T-24 and T-28 treatments resulted in high FCR values while it was negative for T-33 due to the weight loss recorded at that treatment (Table 1). However, once typical growth rates resumed for T-24 and T-28 in the second month, FCR values dropped significantly. The two groups remained comparable in the third month while T-33 exhibited significantly higher values.

**Fig 2:**
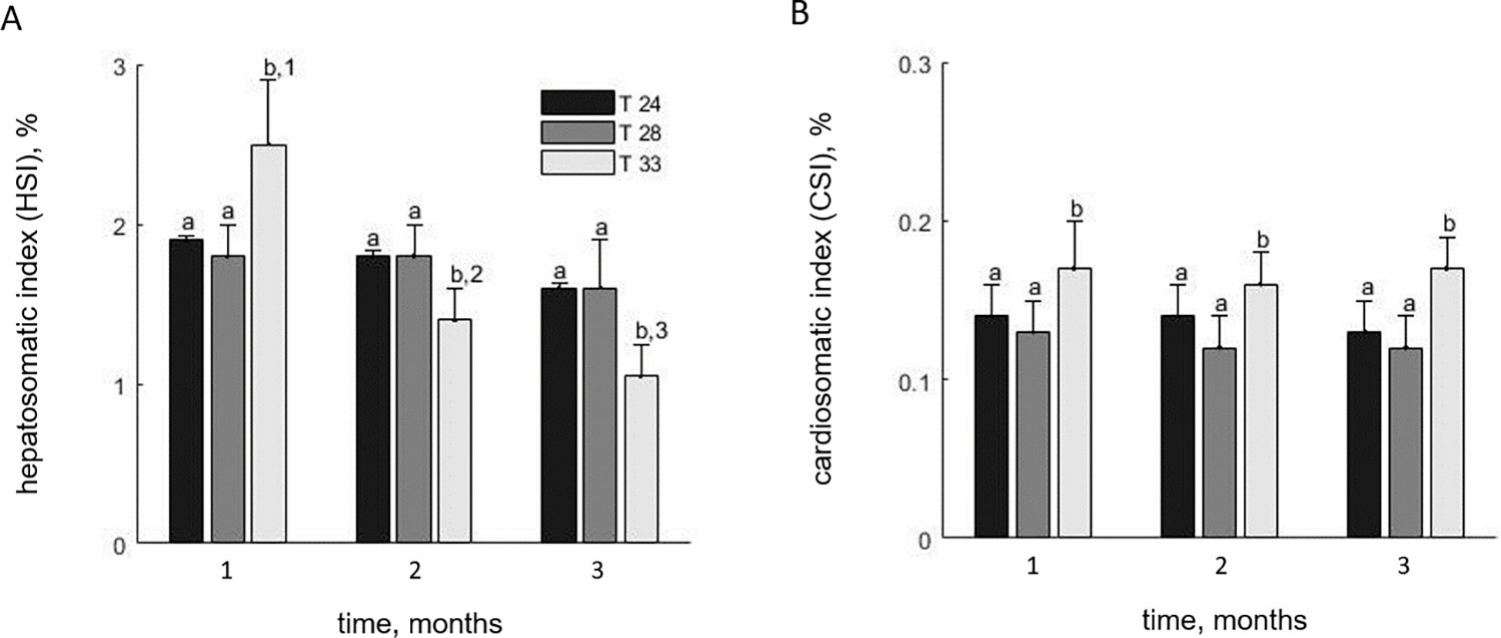
Hepatosomatic (A) and cardiosomatic (B) index for E. sea bass reared under three temperatures (T-24, T-28, T-33). Points denote mean values between replicates and bars the standard deviation (3 replicates, N = 5 per replicate).

With respect to the somatic indices, significant effects on the HSI were found for time and interaction (time: *F_2,108_* = 41.4; *P* < 0.001; interaction: *F_4,108_* = 16.9; *P* < 0.001), as well as a marginally significant effect of temperature (*F_2,108_* = 3.1, *P* = 0.048). There were no statistically significant differences in HSI for the T-24 and T-28 treatments throughout the trial, but differed significantly for T-33, being higher than the other two treatments in the first sampling and lower in the second and third (Fig 2A). The CSI was also affected by temperature (*F_2,108_* = 5.9, *P* = 0.004) while the effects of time and the interaction were not significant (time: *F_2,108_* = 1.1; *P* = 0.367; interaction: *F_4,108_* = 0.9; *P* = 0.418). The index remained conservatively close to 0.13% for T-24 and T-28 during the trial, while on the contrary, fish in T-33 exhibited heart hypertrophy with the CSI being significantly higher in all samplings at approximately 0.17% (Fig 2B).

**Fig 3:**
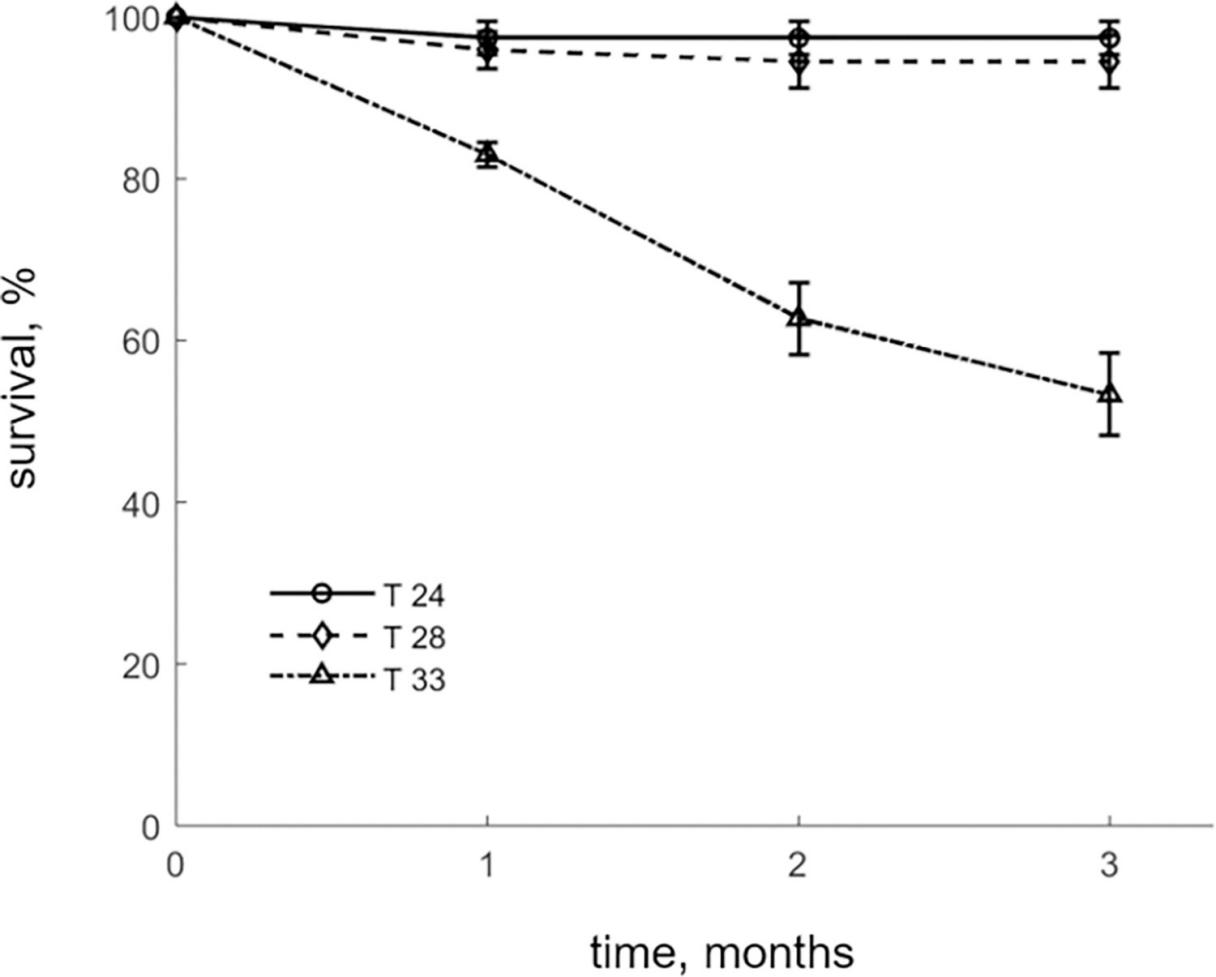
Survival curves for E. sea bass reared under three temperatures (T-24, T-28, T-33). Points denote mean values between replicates and bars the standard deviation.

Finally, a significant effect of temperature on survival was observed as indicated by the one-way ANOVA results in the final month (*F_2,6_* = 28.1; *P* < 0.001). In fact, the lowest and intermediate temperature treatments did not register mortalities throughout the trial apart from a few isolated instances during the first month. However, substantial mortalities were recorded at the highest temperature, with about half of the fish perishing by the end of the experiment as seen by the survival curves (Fig 3). Moreover, on site observations indicated that most of the fish died on days when temperature fluctuated above 33°C, suggesting narrow thresholds for survivability at upper tolerance end.

### Metabolic rate

**Fig 4:**
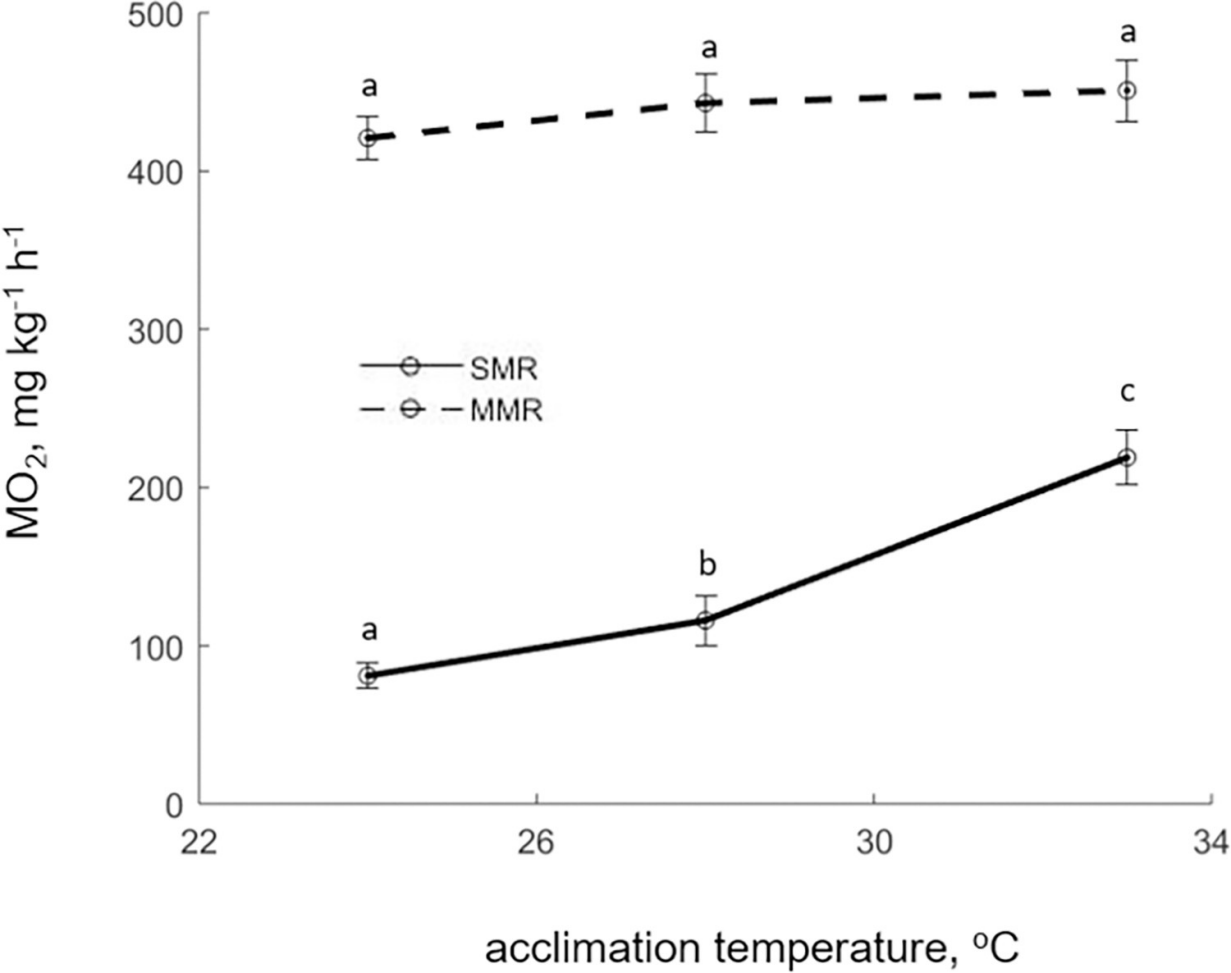
Standard and Maximum Metabolic Rate (SMR and MMR) of E. sea bass at different temperatures. Points represent replicate means and bars the SD (N = 15) and statistically significant differences between treatments are indicated with different letters.

Regarding the metabolic rate analysis, SMR increased substantially with temperature, rising from 81.7 ± 10.4 mg O_2_ kg^-1^ h^-1^ at T-24 to 219.0 ± 17 mg O_2_ kg^-1^ h^-1^ at T-33 (Fig 4) with all treatments differing statistically from each other. While MMR values exhibited the same pattern, the trend was much less pronounced with the increase in oxygen consumption being negligible and no significant differences between the treatments (421.0 ± 13.7, 443.6 ± 16.3, and 451 ± 19.2 mg O_2_ kg^-1^ h^-1^ for the three treatments respectively). This resulted in the aerobic scope, both absolute and factorial, decreasing with temperature. Specifically, the values of the absolute aerobic scope for the three treatments were 340.8 ± 8.9, 327 ± 8.2, and 232 ± 8.6 mg O_2_ kg^-1^ h^-1^, and that of the factorial aerobic scope 5.2 ± 0.6, 3.9 ± 0.4, and 2.1 ± 0.1, respectively.

### Acute thermal challenge

With respect to the acute thermal challenge and the determination of CT_max_, fish became progressively more active as the temperature increased, exhibiting signs of distress a few degrees before reaching the endpoint (Loss of Equilibrium). The endpoint was reached faster as acclimation temperature increased, with a calculated resistance time of 28.1 ± 0.6, 23.8 ± 1.1, and 14.9 ± 0.9 minutes for the three treatments respectively. Acclimation temperature had a strong effect on CT_max_ (*F_2,42_* = 57.6; *P* < 0.001) which increased from 38.05 ± 0.3°C at T-24 to 39.7 ± 0.6°C at T-28 but seemed to plateau afterwards with no significant differences between T-28 and T-33 (40.19 ± 0.5°C). This was also demonstrated by a declining trend in the Acclimation Response Ratio which was calculated at 0.412 for (T-24)-(T-28) and 0.098 for (T-28)-(T-33), thus indicating diminishing gains in acclimation capacity above 28°C. The overall ARR between (T-24)-(T-33) was 0.237.

### Plasma variables

**Fig 5:**
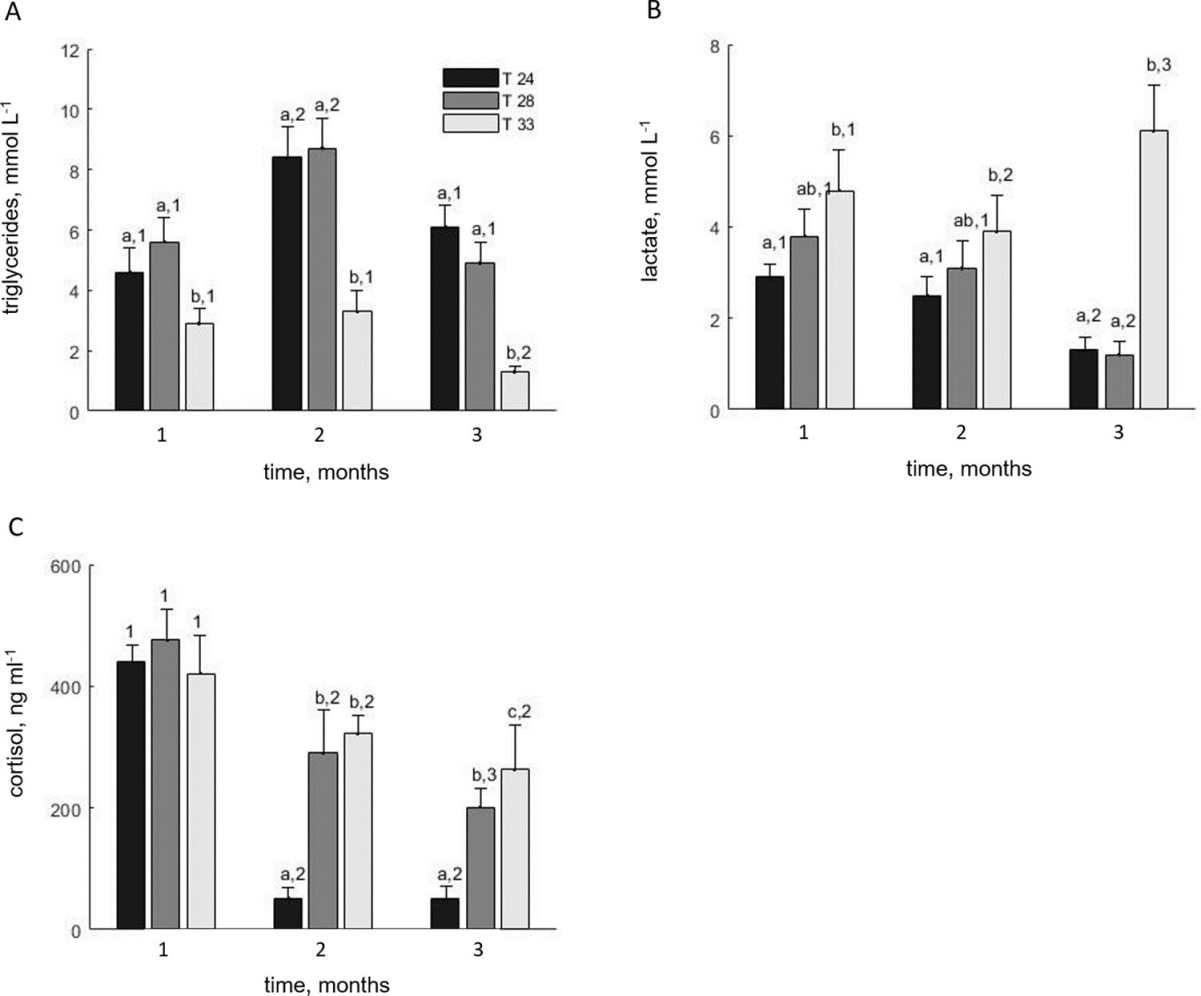
Plasma concentrations of triglycerides (A), lactate (B), and cortisol (C) for E. sea bass reared under three different temperatures (T-24, T-28, T-33). Values represent replicate means and the SD (3 replicates, N = 5 per replicate). Statistically significant differences between treatments in each sampling (month) are indicated with different letters and between samplings for each treatment with different numbers.

Regarding the biochemical variables measured in the plasma, significant temperature, time and interaction effects were found for the triglycerides (temperature: *F_2,108_* = 200.1, *P* < 0.001; time: *F_2,108_* = 140.3; *P* < 0.001; interaction: *F_4,108_* = 35.7; *P* < 0.001). Specifically, the triglycerides concentration differed between T-33 and the other treatments, being statistically lower for the former in the second and third months and reaching values as low as 1.4 ± 0.2 mmol l^-1^ (Fig 5A). Moreover, for the T-24 and T-28 treatments, triglycerides concentration was statistically higher in the second month compared to the other two samplings.

Cortisol levels were also affected by temperature and time (temperature: *F_2,108_* = 84.4, *P* < 0.001; time: *F_2,108_* = 309.3, *P* < 0.001) as well as the interaction (*F_4,108_* = 26.7; *P* < 0.001). In particular, cortisol exhibited elevated values in all treatments during the first month, exceeding 400 ng ml^-1^. However, for T-24, average cortisol values dropped tenfold in the second and third month (52 ± 18 and 50 ± 21 ng ml^-1^ respectively) being significantly lower from the other two treatments (Fig 5C). Although the trend was the same for T-28 and T-33, the degree was less pronounced with cortisol concentrations remaining high (> 200 ng ml^-1^) until the end of the trial.

Similarly for lactate (temperature: *F_2,108_* = 109.2, *P* < 0.001; time: *F_2,108_* = 16.5; *P* < 0.001; interaction: *F_4,108_* = 41.4; *P* < 0.001), all treatments exhibited high concentrations in the first month but were substantially lowered for T-24 and T-28 in the third month (1.2–1.3 mmol l^-1^). On the contrary, T-33 showed significantly higher lactate levels both in the first and third months (Fig 5B).

### Gene expression

**Fig 6:**
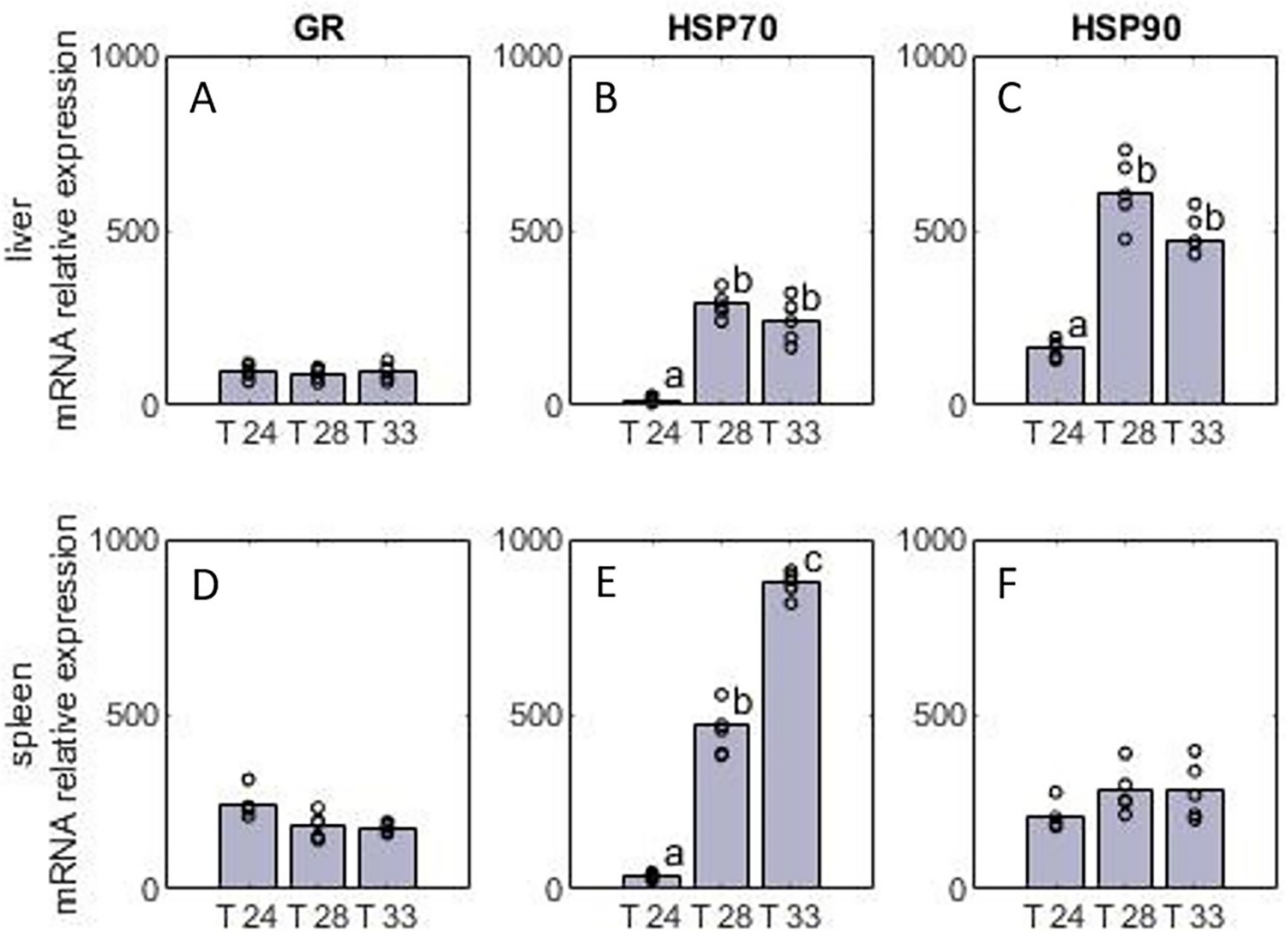
Relative expression of three target genes for E. sea bass reared under three temperatures (T-24, T-28, T-33). A, B, C: the relative expression of GR, HSP70, and HSP90 in the liver. D, E, F: the relative expression of GR, HSP70, and HSP90 respectively in the spleen. Statistically significant differences among treatments are indicated with different letters (one-way ANOVA) while points represent individual measurements (N = 5 per treatment).

The molecular analysis revealed significant effects of temperature in the relative expression of the genes coding for the Heat Shock Proteins HSP70, and HSP90 but not for the GR. Specifically, the effect was significant for HSP70 both in liver (*F_2,12_* = 55.9; *P* < 0.001) and spleen (*F_2,12_* = 411; *P* < 0.001) and for HSP90 in liver (*F_2,12_* = 68.7; *P* < 0.001). In all cases, T-24 exhibited significantly lower values compared to the other treatments. In liver, the pattern was similar for both HSP70 and HSP90 with their expression being upregulated for T-28 and T-33 compared to T-24 (Fig 6B and 6C). While the same trend was observed for HSP90 in spleen, the effect there was not significant. However, the expression of HSP70 in spleen was upregulated substantially for T-28 and even further for T-33 compared to T-24 (by a factor of five and ten respectively).

## Discussion

In this article we present a trial on the responses of E. sea bass to high acclimation temperatures with respect to a number of performance and physiological indicators as well as its tolerance under acute thermal stress. Overall, our results show that while biochemical and molecular biomarkers indicate signs of thermal stress at 28°C, E. sea bass can maintain high whole-animal performance in terms of growth and aerobic scope at that temperature which is encouraging for the species farming in the context of a warming ocean. Moreover, the species shows appreciable capacity to cope with acute thermal stress, especially after acclimation to high temperature regimes, which may indicate resilience to episodes of extreme weather events (heatwaves). However, exposure to temperatures beyond that are not viable for E. sea bass with 33°C being sharply close to the lethal limits as indicated by the poor physiological status, poor performance and high mortality rates.

In nature, European sea bass spends its early life stages in transitional environments (i.e. lagoons) and for that reason it has developed mechanisms to cope with a wide range of temperatures and salinities [62]. However, acclimation to these environmental gradients is a slow process and E. sea bass is known to require substantial time to fully acclimate to new environmental conditions under experimental settings [63]. Both cold and warm acclimation has been reported to last several weeks in fish and this is attributed to thermal acclimation requiring changes in multiple organization levels ranging from fast behavioral and biochemical changes to slow restructuring of tissues and organs such as the gills, the heart and the digestive system [21,64,65]. The growth performance and physiological indicators calculated in this study show indeed that acclimation of E. sea bass to new temperature regimes is a slow process that requires substantial time, perhaps exceeding one month.

With respect to the thermal preferences of E. sea bass, our findings corroborate earlier studies that place the thermal optimum for growth between 24 and 28°C [27,44]. Specifically, in our trial, growth performance was appreciable at both 24 and 28°C, with no substantial differences between the groups in terms of growth and FCR. Indicatively, as reported by other studies, the growth of juvenile E. sea bass when reared in marine cages is the fastest during the summer months (20–25°C) [43] while experiments under controlled temperatures have shown faster growth at 25°C compared to 15, and 20°C [62]. Our study showed a dramatic deterioration of the overall biological performance for the highest temperature treatment. Based on the notion that performance is highest for a narrow range of optimum temperatures beyond which it declines, the observation that growth performance did not differ between the first two treatments indicates that the temperatures of 24 and 28°C lay bilaterally to the temperature optimum. This comes in parallel with the findings of Person-Le Ruyet *et al*. [44] who reported maximum growth for juvenile E. sea bass at 26–27°C. Regarding the highest temperature treatment, the analysis showed poor overall health, diminished performance and signs of chronic thermal stress. Appetite was significantly reduced, and growth ceased, reflecting a high energetic cost at 33°C, mortality was high, and the thresholds for survivability appeared to be particularly narrow around 33°C. Similar decline in growth performance indicators has been reported for smaller E. sea bass (5 g) reared for one month at 32°C [46], suggesting that the upper performance limits for the species may be size-independent at this size range. Furthermore, there was a shift in somatic indices. The HSI was lower than the other treatments, similarly to what Islam *et al*. [46] report at 32°C compared to 24°C. In addition, fish exhibited heart hypertrophy which is a phenomenon documented in fishes as an adaptive response to elevated temperatures aiming to increase the cardiovascular output [66,67] but has not been previously reported for E. sea bass. Although moderate heart remodeling may not always be considered pathological since it is naturally occurring in some fish populations during the winter [35,68], it is generally interpreted as a product of chronic anemia which imposes biomechanical stress to the heart [69]. Moreover, heart hypertrophy has been positively correlated with cortisol response to stress in salmonids [70]. Therefore, the observation that fish in the present study exhibited heart hypertrophy after only one month may be indicative of high chronic stress levels. While the phenomenon has not been adequately studied in fish in relation to temperature [71], our findings may point to heart hypertrophy as an early sign of distress that should be considered when addressing welfare issues in the context of climate change.

The metabolic rate analysis also highlighted the diminished capacity of E. sea bass at the highest temperature treatment. Specifically, energetic costs increased with temperature causing a substantial increase of SMR from 24 to 33°C in a fashion consistent with literature [30,45,72]. Indicatively, Claireaux and Lagadere [73] recorded similar SMR values of 91 mg O_2_ h^-1^ kg^-1^ at 25°C, the highest temperature tested in that study. However, the maximum metabolic rate did not increase similarly but rather seemed to plateau at 28°C, with no further increase at 33°C. This complements the observations of Claireaux *et al*. [45] who did not record significant increase of MMR between 26–30°C, indicating a limited capacity for E. sea bass to further increase its cardiovascular output beyond those temperatures. As a result, the aerobic scope, both absolute and factorial, was the lowest at 33°C, suggesting a close proximity to the upper end of the thermal tolerance range for E. sea bass. Interestingly, while significant mortalities occurred at 33°C, the fish maintained some aerobic capacity since their aerobic scope was far from zero. This suggests that thermal tolerance in E. sea bass may not be solely interpreted by oxygen limitations but also influenced by other mechanisms worthy of further investigation as suggested elsewhere [74].

With respect to the thermal limits of E. sea bass under acute stress, it appears that the temperature history of the animals plays a crucial role. This was demonstrated here by the shift of CT_max_ under different acclimation temperatures. For E. sea bass farmed in northern France and acclimated to 13°C, a CT_max_ of 33°C has been recorded [47] while trials conducted in the 15–25°C range report a CT_max_ of 36.7°C for fish reared at 25°C [62]. Here, we report CT_max_ as high as 40°C for fish acclimated at 33°C, which could be attributed to the high acclimation temperature but also to the genetic origin of the fish (Eastern Mediterranean). The genetic background is known to affect the responses of E. sea bass to its environment including pathogen resistance [75] and swimming performance [76]. It is likely that the animals used in this study differ in their thermal capacity compared to those of Western Mediterranean or Atlantic origin and it would, therefore, be interesting to study the thermal responses of different populations comparatively in the future. In turn, this may contribute to assisted evolution efforts in E. sea bass through breeding, conditioning, and epigenetic programs which aim to improve the thermal tolerance traits of the fish and have been recognized as indispensable tools for mitigating the effects of climate change [77]. Admittedly, CT_max_ is sensitive to the protocol used since it is a time-dependent tolerance measurement and caution should be exercised for comparison between studies [78]. That being said, the heating rate, which is arguably the most defining parameter for CT_max_, does not seem to significantly affect the measurement on E. sea bass, at least for the larval stages [38]. Moreover, the Acclimation Response Ratio, an index used to study the magnitude of thermal acclimation, provides insights into the species-specific thermal strategies of E. sea bass. As shown by changes in the ARR in this study, the acclimation capacity declined at high temperatures with the ARR decreasing from 0.41 between 24–28°C to 0.09 between 28–33°C, and with an overall value of 0.24 for the 24–33°C range. For comparison, Dülger *et al*. [40] similarly report ARR values of 0.25–0.27 for acclimation temperatures between 15–25°C. Such values are comparable to some subtropical species [79] which are exposed to large temperature fluctuations, much like E. sea bass is during its early life stages. Therefore, it appears that, in general, the species has appreciable capacity to cope with acute thermal stress which is encouraging in the context of climate change and future heatwave events. Yet, acclimation to temperatures exceeding 28°C shows diminishing returns on increasing tolerance, thus, also pointing to the capacity limits for the species.

Apart from improving our understanding of the species thermal limits, the above observations may point to potential practical uses for aquaculture with respect to tolerance gains related to acclimation. Admittedly, thermal acclimation under commercial farming conditions is not feasible because the fish are predominantly reared in marine cages where there is limited control over the environmental conditions. However, there is evidence that acclimation to high temperatures during larvae rearing and nursing not only affects the growth performance at later life stages [42] but may also result in increased thermal tolerance [77,80]. From a farming perspective this is worth of further investigation for E. sea bass since in our study, the fish showed appreciable capacity for thermal acclimation. Another potential utilization of these findings could be related to site selection. The species is predominantly farmed in coastal areas which generally exhibit high temperature fluctuations compared to offshore areas and are therefore more vulnerable to heatwaves [81] Among other benefits of expanding the aquaculture activity offshore [81–83], selection of sites with lower temperature fluctuations could provide sufficient acclimation time for E. sea bass to increase its thermal tolerance and therefore, its resilience to climate change.

The physiological indicators determined for the 24 and 28°C treatments indicate signs of thermal stress at the latter temperature. Specifically, at 24°C cortisol levels dropped dramatically after the first month and remained low until the end at typical baseline values for the species [28,84]. Lactate levels also decreased over time while other metabolites such as triglycerides increased in the second month but remained within the typical range of E. sea bass [27,85,86]. The temporal pattern of these indicators confirms that thermal acclimation of E. sea bass is a slow process that lasts several weeks while also highlighting the need for long term studies on the thermal response of the species. Moreover, it shows that an array of indicators should be used in conjunction for elucidating thermal responses since the above indicators appeared suitable for detecting early signs of thermal stress as opposed to growth performance and metabolic rate which provided information on long-term acclimation responses. The trend was similar at 28°C with cortisol and lactate decreasing after the first month but to a lesser degree. The presence of relatively high cortisol concentrations until the end of the trial may signify signs of stress at that temperature, and in fact, such high baseline cortisol levels that exceed 200 ng ml^-1^ have also been reported for E. sea bass during the warm summer months (25–28°C) in the Mediterranean [28]. This was further supported by the upregulation of heat shock proteins compared to 24°C, which is indicative of the activation of protective cellular mechanisms [87]. At the highest temperature treatment, stress levels, in term of cortisol, lactate, and HSP expression remained high after the first month until the end of the trial while the low triglycerides concentrations suggest that the fish had diminished capacity for metabolic upregulation. Similar increase of HSP, cortisol and lactate have been reported for E. sea bass fingerlings at 32°C [26,27] as well as other species reared under high temperature regimes such as the gilthead seabream [88], the largemouth bass (*Micropterus salmoides*, Lacepède, 1802) [89], the South American trout (*Brycon amazonicus*, Spix and Agassiz, 1829) [90], the meagre (*Argyrosomus regius*) [91] and the rainbow trout (*Oncorhynchus mykiss*, Walbaum, 1792) [92]. Therefore, as indicated by the upregulation of HSP, the depression of metabolites such as triglycerides and the high levels of stress biomarkers, the cellular machinery of the animals was on the verge of collapse, suggesting proximity to the lethal chronic temperature limits for the species.

The array of performance, metabolic, biochemical, and genetic indicators analyzed in this study, offer insights into the responses of E. sea bass to high acclimation temperatures as well as its tolerance under acute thermal stress. This is critical for the aquaculture industry in the context of climate change since appropriate management and climate mitigation strategies must rely on sound biological grounds. Enhancing our understanding on the optimal temperatures and the tolerance thresholds for a species, which this study strives to do, could contribute not only to decisions regarding site selection as mentioned previously but also to altering management practices such as the timing of seeding and the harvesting size in order to optimize production at a given area [4,6,8]. For instance, adjusting the time of seeding may allow certain areas to benefit from the prolonged warmer season in terms of growth [4] while areas where summer temperatures are already close to the thermal limits reported here may benefit from a harvesting strategy towards smaller sizes, thus, minimizing losses due to heatwaves. Moreover, while specific recommendations cannot be directly drawn from our results due to being highly site-specific and requiring the development of appropriate tools such as models, maps, risk and opportunity analyses, and decision support systems, the biological responses of E. sea bass to elevated temperatures reported here may in fact be highly valuable in developing, parametrizing, and refining such tools.

## Conclusions

The metabolic responses and overall performance of E. sea bass under high acclimation temperatures as well as its tolerance to acute thermal stress are critical due to the species commercial value and the future uncertainty surrounding climate change. Under prolonged (three months) exposure to 24, 28, and 33°C, which is generally lacking from literature and especially for commercial size fish, the present trial points to the 24–28°C range as the optimum for the species performance corroborating previous studies. Moreover, while biochemical and molecular biomarkers indicate signs of thermal stress at 28°C, E. sea bass is able to maintain high performance at that temperature which is encouraging for the species farming in the context of a warming ocean. Critical survival thresholds appear sharply close to 33°C, where whole-animal performance diminishes and the fish exhibit poor physiological status. Fish at the highest temperature exhibited heart hypertrophy (not previously documented for E. sea bass) in an attempt to increase their cardiovascular output. Yet, this did not translate to increase on the Maximum Metabolic Rate which reached a plateau above 28°C. Regardless, at the highest temperature fish exhibited the lowest aerobic scope which is reflective of their diminished performance. Finally, the species shows appreciable capacity to cope with acute thermal stress, especially after acclimation to high temperature regimes, which may indicate resilience to episodes of extreme weather events (heatwaves). However, this capacity has diminishing tolerance returns for acclimation beyond 28°C highlighting the limitations for the species.
